# Reappraisal of incentives ameliorates choking under pressure and is correlated with changes in the neural representations of incentives

**DOI:** 10.1093/scan/nsy108

**Published:** 2018-11-27

**Authors:** Simon Dunne, Vikram S Chib, Joseph Berleant, John P O’Doherty

**Affiliations:** 1 Computation and Neural Systems, California Institute of Technology, Pasadena, CA, USA; 2 Division of the Humanities and Social Sciences, California Institute of Technology, Pasadena, CA, USA; 3 Department of Biomedical Engineering, Johns Hopkins University, Baltimore, MD, USA

**Keywords:** choking, ventral striatum, skin conductance, reappraisal

## Abstract

It has been observed that the performing for high stakes can, paradoxically, lead to uncharacteristically poor performance. Here we investigate a novel approach to attenuating such ‘choking under pressure’ by instructing participants performing a demanding motor task that rewards successful performance with a monetary gain, to reappraise this incentive as a monetary loss for unsuccessful performance. We show that when participants applied this simple strategy, choking was significantly reduced. This strategy also influenced participants’ neural and physiological activity. When participants reappraised the incentive as a potential monetary loss, the representation of the magnitude of the incentive in the ventral striatum Blood Oxygenation Level Dependent (BOLD) signal was attenuated. In addition, individual differences in the degree of attenuation of the neural response to incentive predicted the effectiveness of the reappraisal strategy in reducing choking. Furthermore, participants’ skin conductance changed in proportion to the magnitude of the incentive being played for, and was exaggerated on high incentive trials on which participants failed. Reappraisal of the incentive abolished this exaggerated skin conductance response. This represents the first experimental association of sympathetic arousal with choking. Taken together, these results suggest that reappraisal of the incentive is indeed a promising intervention for attenuating choking under pressure.

## Introduction

Many significant moments in life, such as academic examinations and athletic competitions, require the demonstration of a skill with the promise of great social or material reward for successful performance. However, although moderate incentives may facilitate successful performance (Prendergast, 1999), high incentives can have deleterious effects on performance (Baumeister, 1984). This paradoxical reduction in performance when incentives are high is referred to as ‘choking under pressure’ (Beilock and Carr, 2001; Dandy *et al*., 2001; Beilock and Carr, 2005; Mobbs *et al*., 2009; Pope and Schweitzer, 2011; Chib *et al*., 2012, 2014; Lee and Grafton, 2015). Given the pernicious effects of choking on performance, there has been considerable interest in developing psychological interventions that mitigate it (Beilock and Carr, 2001; Ramirez and Beilock, 2011; Balk *et al*., 2013).

Previous work from our group has investigated the extent to which the nature of the incentive influences choking under pressure. In particular, we found (Chib *et al*., 2012, 2014) that the level of choking in a challenging motor task depended on both whether participants were performing to obtain a monetary gain or to avoid a monetary loss, as well as their aversion to monetary losses relative to monetary gains (Kahneman and Tversky, 1979). Specifically, Chib *et al*. (2012, 2014) reported that when participants were playing to win money, those who were highly loss averse showed performance decrements when the monetary incentive was high. However, when playing to avoid monetary losses, the performance of such highly loss-averse participants did not suffer when incentive was high (Chib *et al*., 2014). Furthermore, in those studies, activity in the ventral striatum correlated with choking under pressure.

These results suggest that customizing the incentive structure an individual faces may avert performance decrements at high incentives. However, in many real-world situations, it is not feasible to tailor the incentive structure to the individual; for example, a professional athlete is unlikely to find the prospect of avoiding monetary losses an attractive incentive to play. Thus, an applicable technique for overcoming choking that is based on these findings should not require the manipulation of the external incentive structure. One possibility may be to apply a cognitive strategy called reappraisal, a form of emotional regulation in which individuals reinterpret the affective meaning of a stimulus in order to moderate the emotional response that is subsequently engendered (Gross, 2002; Webb *et al*., 2012).

The present study had multiple goals; firstly, we investigated whether it is possible to directly influence the behavioral susceptibility of participants to choking by explicitly instructing them to reappraise an incentive. In our paradigm, participants performed a challenging motor task in order to win money. In the ‘baseline’ condition, participants were instructed to regard the incentive as it was—a monetary gain for successful performance. However, when playing under the ‘reappraisal’ condition, participants were instead asked to reappraise the incentive as a monetary loss for unsuccessful performance. Given our prior findings that individual differences in behavioral loss aversion moderate the influence of incentives on choking under pressure (Chib *et al*., 2012, 2014), we hypothesized that individual differences in the behavioral susceptibility to loss aversion would interact with the reappraisal manipulation in order to influence choking; specifically, those high in loss aversion would choke at high levels of incentive when playing for a monetary gain, but not when reappraising the incentive as the avoidance of a potential monetary loss.

Our previous work (Chib *et al*., 2012, 2014) suggests that the ventral striatum is a key component of the neural circuits involved in determining choking effects. Thus, a second goal of this study was to investigate whether activity in the ventral striatum would be directly modulated by the reappraisal manipulation. Specifically, if the behavioral effects of reappraisal truly derive from an internal manipulation of the incentive, we predicted that this would be reflected in differential neural encoding of the incentive in the reappraisal compared to the baseline condition within this brain region. To test these predictions, we scanned participants with functional magnetic resonance imaging (fMRI) while they performed the incentivized motor task.

A final goal of the study was to investigate the psychophysiological mechanisms underpinning choking. It has been noted (Ariely *et al*., 2009; Balk *et al*., 2013) that the initial improvement and subsequent decrement of performance with increasing incentive is reminiscent of the bell-shaped relationship between performance and arousal described by the `Yerkes–Dodson law’ (Yerkes and Dodson, 1908). In spite of the long history of this association, surprisingly little evidence has been accumulated to directly support the claim of a relationship between choking and sympathetic hyperarousal. Consequently, we also measured skin conductance in our participants while they were performing the behavioral task in the scanner. We hypothesized that if sympathetic arousal levels are associated with choking behavior, we should find evidence of increased arousal during task performance in the experimental condition in which participants exhibit choking.

If we can find evidence that choking behavior can be moderated via a simple reappraisal strategy, accompanied by supporting evidence from both psychophysiological and neural data, this would potentially open up new avenues for the development of psychological interventions for mitigating the effects of choking in real world situations.

## Methods

### Participants

Forty-two participants (mean age 27 years, age range 18–49 years, 16 females) took part in the experiment. All participants were right-handed and were prescreened to exclude those with a previous history of neurological or psychiatric illness. The California Institute of Technology Institutional Review Board approved this study, and all participants gave informed consent. Four participants who participated in the initial training phase did not complete the neuroimaging phase of the experiment due to scheduling conflicts (2) and illness (2), and were excluded from the analysis. We report the results of skin conductance analyses of the remaining 38 participants. Two participants with extreme loss aversion values (0, 10) were excluded from behavioral analyses involving loss aversion, leaving *n* = 36. Two participants were excluded from the MR imaging analysis due to excessive head motion (>3 mm in any direction) during imaging, leaving *n* = 36.

### Procedure

**Fig. 1:**
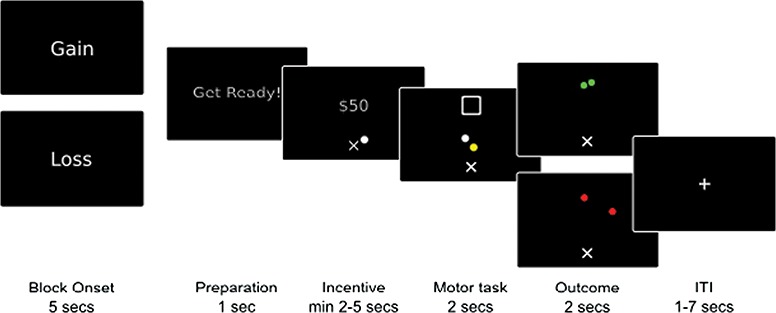
Task schematic. Participants played alternating blocks of baseline and reappraisal trials, with the block type indicated at the beginning of each block. Each trial began with the presentation of the monetary incentive the participant was playing for ($0, $25, $50, $75, $100). To begin the motor task, participants held a white cursor that represented the position of their finger in space over the starting position (‘x’) for a randomly varying duration (2–5 s). When the target square appeared, participants had 2 s to bring the white finger cursor and a yellow cursor, which moved as if it were a mass attached to the white cursor by a spring, to rest inside the square. At the end of the trial participants saw whether they had been successful (green cursors) or unsuccessful (red cursors). Trials were followed by an intertrial interval (1–7 s).

Each participant attended the experiment on two separate days. On the first day, participants began by completing the prospect theory gambling task (see Supplementary Materials ), which was used to measure their level of loss aversion. Participants were then introduced to the motor task, in which they controlled a virtual spring-mass system. This was a modified version of the task developed by us (Chib *et al*., 2012, 2014) to investigate choking and is described fully in the Supplementary Materials. This dynamic system was completely novel to the participants, which allowed us to evaluate their performance uncorrupted by previous experiences or expertise. On that day, participants learned to perform the motor task (training phase), after which we determined participants’ rates of success at various target sizes (thresholding phase). Both the training and thresholding phases took place in a mock MRI scanner to replicate the posture necessary for the scanning environment. After this, participants received instruction in the cognitive reappraisal strategy they would implement during the motor task (see next section). They then performed the motor task for money (see Figure 1) while undergoing MRI and having their skin conductance recorded (testing phase). Following the scan, participants completed a debriefing in which they indicated the level of performance they believed they had achieved at each incentive level under the baseline and reappraisal conditions, and how successful they believe they had been in applying the reappraisal strategy on a 1–10 rating scale. Participants were paid a fee of $35 plus their earnings from the gambling task and the outcome of a single randomly selected trial from the testing phase of the motor task.

### Reappraisal strategy

In the study we instructed participants in a cognitive reappraisal strategy, which is a form of emotion regulation that aims to change the trajectory of an emotional response by reinterpreting the meaning of the emotional stimulus (Ray *et al*., 2010). In this context, the emotional stimulus was the monetary incentive that participants played for on each trial.

This reappraisal intervention differs from reframing, a manipulation which we have employed in a previous study of choking (Chib *et al*., 2014), where the monetary incentive was presented by the experimenter as a monetary loss rather than a monetary gain. In contrast, in the present study the incentive was presented identically in both the baseline and reappraisal conditions. What differed between the conditions here was the interpretation applied by the participant in the reappraisal condition.

Specifically, participants were instructed to interpret the monetary incentives presented during the motor task differently based on the prompt that appeared before each block of trials.

In the baseline condition participants were instructed to interpret the incentive as a potential monetary gain, while in the reappraisal condition participants were asked to imagine the incentive as a potential monetary loss. These instructions cued participants to explicitly imagine the financial and emotional consequences of their success or failure under each condition. The instructions provided to participants read as follows:

`During the session you will see the word Loss and the word Gain appear onscreen. We would like you to think about the monetary incentives in different ways when you see these words.

When the word Loss appears on screen (see image below), you should regard the monetary incentives shown at the beginning of each round as “your” money. Imagine the amount, in cash, sitting in your pocket as you complete the round. Imagine that, if you are successful on the round, you will get to keep your money, but if you are unsuccessful, you will have to give this money to the experimenter. Imagine how it would feel to lose this money. You should continue to think about the incentives in this way throughout each round until you see the word Gain appear on screen.

When the word Gain appears on screen (see image below), you should imagine that you begin each round with no money in your pocket. Regard the monetary incentive as an amount of money that you have the opportunity to win. Imagine that if you are successful on the round, the experimenter will give you this money, in cash, but if you are unsuccessful you will end the round as you began—with nothing. Imagine how it would feel to gain this money. You should continue to think about the incentives in this way throughout each round until you see the word Loss appear on screen.

Please do your best to think of the incentives in these ways throughout the session.’

When participants had read these instructions, they were explicitly reminded by the experimenter that although they should interpret the incentive differently in the baseline and reappraisal conditions, in reality the incentives on all trials would be treated as potential monetary gains. That is, successful performance in the randomly selected trials would result in a monetary gain for the participant, while failure would result in no change in their earnings from the task. Participants did not undergo any further training in the reappraisal strategy before performing the task.

### MRI protocol

Magnetic resonance imaging was carried out with a 3T Siemens Trio scanner and radio frequency coil. High-resolution structural images were collected using a standard magnetization-prepared rapid gradient-echo (MP-RAGE) pulse sequence, providing full brain coverage at a resolution of 1 × 1 × 1 mm. Functional images were collected at an angle of 30° from the anterior commissure–posterior commissure axis, to attenuate signal dropout in orbitofrontal cortex (Deichmann *et al*., 2003). Forty-five ascending slices were acquired at a resolution of 3 × 3 × 3 mm, providing whole-brain coverage. A one-shot echo-planar imaging pulse sequence was used (TR, 2800 ms; TE, 30 ms; FOV, 100 mm; flip angle, 80°). Participants completed three functional runs with a maximum duration of 22.4 min each.

### Behavioral analysis

In order to measure the degree to which a participant choked, we calculated for each condition (baseline and reappraisal) the proportion of successful trials at each incentive level ($0, $25, $50, $75, $100) of a given participant. The choking metric in a particular condition was then the difference between that participant’s average performance at the incentive level at which it peaked and the participant’s average performance at the highest level of incentive ($100). This quantity took a value of zero if performance peaked at the highest level of incentive ($100), while when performance peaked at lower levels of incentive this metric is necessarily greater than or equal to zero. High values on this metric indicate that participant’s performance at the highest level of incentive was substantially worse than that at its peak. Unlike a simple comparison of performance at high and medium incentive levels, this metric contains full quantitative information about the deterioration of performance from its peak when faced with very high incentive. In addition, it also accommodates individual differences in the incentive level at which participants may begin to choke. Therefore, we believe it represents a more sensitive and appropriate method for detecting and measuring choking, than a comparison of performance at high and medium incentive levels.

This choking metric is continuously distributed over a range of values but takes one focal value, zero, with positive probability. The application of ordinary least squares regression to such a variable is known to yield inconsistent parameter estimates (Amemiya, 1973). Therefore, Tobit regression (Tobin, 1958), which accommodates such data, was used to regress the choking metric on appraisal strategy (baseline = 0, reappraisal = 1) and mean-corrected loss aversion, with a random participant-level intercept, and was implemented using the AER package (Kleiber and Zeileis, 2008) in R (R Core Team, 2015). The Tobit describes the relationship between an observable dependent variable $y$ (here, the choking metric), independent variables ${x}_j$ (here, appraisal strategy, loss aversion and an intercept term) and an intervening unobservable latent variable ${y}^{\ast }$ as taking the following form:

$$ {y}_i=\left\{\begin{array}{c}{y}_i^{\ast}\\ {}0\end{array}\kern1.5em \genfrac{}{}{0pt}{}{if\ {y}_i^{\ast }>0}{if\ {y}_i^{\ast}\le 0,}\right. $$

where ${y}_i^{\ast }=\sum_j{\beta}_j{x}_{i,j}+{u}_i,\kern0.5em {u}_i\sim N(0,{\sigma}^2)$. We calculated the partial effect of reappraisal on choking as the difference in the expected magnitude of choking between the baseline and reappraisal strategies for an individual of mean loss aversion, where $E(y|x)=\Phi \big(\frac{\sum_j{x}_j{\beta}_j}{\sigma}\big){\sum}_j{x}_j{\beta}_j+\sigma \phi \big(\frac{\sum_j{x}_j{\beta}_j}{\sigma}\big)$, (Wooldridge, 2010).

### MRI preprocessing

All image preprocessing and analysis was performed using SPM12 (Wellcome Department of Imaging Neuroscience, Institute of Neurology, London, UK; available at http://www.fil.ion.ucl.ac.uk/spm). All functional volumes were corrected for differences in acquisition time between slices (to the middle slice), realigned to the first volume and coregistered with the high-resolution structural image. The coregistered high-resolution structural image was segmented and normalised to Montreal Neurological Institute space using Diffeomorphic Anatomical Registration Through Exponentiated Lie algebra (DARTEL). The resulting transformation was applied to the functional volumes. The functional volumes were spatially smoothed with a Gaussian kernel (full-width at half-maximum = 6 mm) and high-pass temporally filtered (128 s). The description of the fMRI statistical analysis can be found in the Supplemental Materials.

### Skin conductance analysis

Skin conductance was recorded on the thenar/hypothenar surface of the left hand using Ag/AgCl radio-translucent electrodes (EL509; Biopac Systems Inc., Holliston, MA), 0.5%-NaCl electrode paste (GEL101; Biopac) and MR-compatible leads (LEAD108C). The signal was acquired with a Biopac data acquisition system (modules EDA100C-MRI and MP150) connected to the stimulus presentation computer. A model-based analysis of the skin conductance data was conducted using the PsPM toolbox (Bach and Friston, 2013) for MATLAB. This permits the inference of effects of experimental events on sympathetic nerve firing by inverting an informed generative model of skin conductance, and has been demonstrated to have favorable predictive validity relative to alternative analysis techniques (Bach, 2014). The data was filtered with a unidirectional Butterworth band pass filter with cut-off frequencies of 0.05 and 5 Hz, and downsampled from the acquisition frequency of 100 Hz to 5 Hz. For each participant, this data was modeled using a general linear convolutional model with participant-specific design matrices. We created three boxcar onset regressors representing the periods of incentive presentation (2–5 s), motor task (2 s) and intertrial interval (1–7 s), parametric regressors at the time of incentive presentation and motor task representing task performance (success = 1, failure = 0), condition (reappraisal = 1, baseline = 0) and incentive magnitude, as well as regressors representing the two- and three-way interactions of these variables. These regressors were convolved with a canonical skin conductance response function and its first temporal derivative (Bach *et al*., 2010) and included in the design matrix. Group effects were determined by entering the parameter estimates for each convolved regressor in between-subjects two-tailed *t*-tests.

## Results

### Choking is reduced by reappraisal

We predicted that participants’ performance would be influenced by the magnitude of the incentive, the appraisal strategy and their individual level of loss aversion. We predicted that at the highest level of incentive the performance of highly loss-averse participants would deteriorate when they interpreted the incentive as a monetary gain. Furthermore, we predicted that this deterioration would be ameliorated by reappraisal of the incentive as a loss.

We began by operationalizing choking as the difference between a participant’s peak level of performance across incentives and their level of performance at the highest level of incentive, calculated separately for the baseline and reappraisal conditions. This allowed us to account for the fact that the incentive at which performance peaked varies across participants. A participant whose performance peaked at $100 would necessarily have a choking value of 0 according to this metric, while a participant whose performance peaked at a lower level of incentive would have a positive value on this metric (see Supplementary Figure S1 for a histogram of these values).

To determine whether choking occurred in the baseline condition and whether participants’ cognitive reappraisal of the incentive influenced their likelihood of choking, we regressed this metric on appraisal condition (baseline = 0, reappraisal = 1), participant’s level of loss aversion and their interaction (see Supplementary Table S1). We found that choking in the baseline condition was significantly greater than zero $[\beta (SE)=14.23(7.09),\kern0.5em P<0.04].$ This demonstrates that in the baseline condition where participants interpreted the incentive as a potential monetary gain, performance at the highest level of incentive was indeed significantly worse than performance at its peak.

**Fig. 2:**
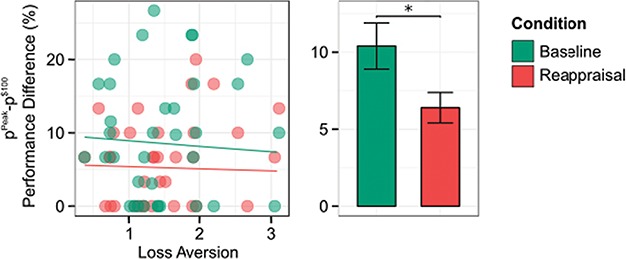
Participant behaviour. Choking was operationalized as the difference between peak performance and performance at the highest level of incentive ($100). Choking was not reliably associated with loss aversion (left), but was significantly greater in the baseline condition when participants appraised the incentive as a monetary baseline (right). Box centers correspond to median values, box bottom and top correspond to the first and third quartiles, respectively, and whiskers represent the maximum and minimum values. ^*^ indicates significant at *P* < 0.05.

In addition, we found that this drop in performance was significantly attenuated when participants applied the reappraisal strategy $[\beta (SE)=-5.74(2.62),P=0.03]$. See Figure 2 for an illustration of the individual levels of choking for each participant in each condition, as well as the average across participants in each condition. That is, reappraising the gain as a potential loss resulted in a significant decrement in choking across participants; for a participant with average loss aversion, the expected difference between performance at its peak and performance at the highest level of incentive when the incentive was regarded as a monetary gain was 6.70%, while reappraisal of the incentive as a monetary loss reduced this to 1.5%. This effect remained significant when regressors with statistically insignificant effects were dropped from the model (see Supplementary Table S1). In a follow-up regression analysis of participants success rates we find no average difference in success rates between the baseline and reappraisal conditions, and no difference between the conditions in the average effect of incentive on success rates (see Supplementary Results). For an illustration of average performance at low, medium and high incentive see Supplementary Figure S3.

### Interaction effect between appraisal and loss aversion on choking

Our main behavioral hypothesis was that the appraisal strategy and loss aversion would interact to influence choking. Unexpectedly, the Tobit regression revealed no such interaction of loss aversion and appraisal strategy. The effect of loss aversion on choking in the baseline condition was not statistically significant $[\beta (SE)=-1.03(1.13.),\kern0.5em P=0.36]$, nor was the effect of loss aversion during reappraisal significantly different from observed that in the baseline condition $[\beta (SE)=0.51(1.79),\kern0.5em P=0.77$]. We also find no effect of participants’ self-assessed ability to reappraise, which they provided after completing the task $[\beta (SE)=-0.82\ (0.90), P=0.40]$ (see Supplementary Figure S2 for a histogram of these values).

### Whole brain analysis: neural effects of incentive at the time of incentive presentation

**Fig. 3:**
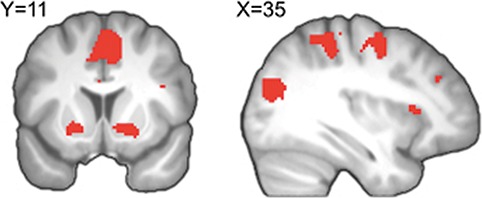
Voxel-wise effects of incentive magnitude on BOLD. During the presentation of the incentive, we observe effects of the magnitude of the incentive magnitude in the baseline and reappraisal conditions in ventral striatum, and a number of cortical areas. For the purpose of illustration, activations are shown corrected for multiple comparisons at pFWE <0.05.

Given that choking is driven by incentive, with choking being a monotonically increasing function of incentive level, it is essential to understand participants’ neural representation of incentive during the task (see Supplementary Table S2). We found that on average across the baseline and reappraisal conditions, BOLD activity during the time of incentive presentation in the left [k = 81; x,y,z = −16,8,−8; t(37) = 7.17, p_FWE_ < 0.05 SVC] and right [k = 79; x,y,z = 20,4,−12, t(37) = 7.42, p_FWE_ < 0.05 SVC] ventral striatum, an extensive frontoparietal cluster [k = 4863; x,y,z = −38,−16,48, t(37) = 11.19, p_FWE_ < 0.05] encompassing lateral prefrontal cortex [k = 82; x,y,z = 32,22,8; t(37) = 7.75, p_FWE_ < 0.05], supplementary motor area and motor cortex [k = 179, x,y,z = 16,−28,66; t(37) = 9.34, p_FWE_ < 0.05], and parietal cortex [k = 1740; x,y,z = 26,-58,62; t(37) = 9.38, p_FWE_ < 0.05] increased with the magnitude of the incentive (Figure 3). These findings are consistent with the results of Chib *et al*. (2012, 2014), who found that encoding of real monetary gains and real losses resulted in positive activation of the ventral striatum and a network of frontoparietal areas.

### Whole brain analysis: effects of incentive at the time of motor task performance

During the time of performance of the motor task, we observe effects of the incentive magnitude on the BOLD signal in middle temporal [k = 6; x,y,z = −34,−60,10; t(37) = 6.18, p_FWE_ < 0.05] and bilateral precentral gyrus [k = 9; x,y,z = −32,−28,−4; t(37) = 6.04, p_FWE_ < 0.05; see Supplementary Table S2]. However we do not observe effects of the incentive magnitude in the ventral striatum during this period. We found no significant differences between the baseline and reappraisal conditions in the BOLD response to incentive magnitude during the period of motor task performance.

### Region of interest analysis: neural response to incentive in ventral striatum

**Fig. 4:**
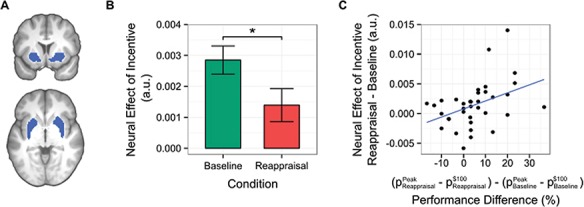
Effects of incentive magnitude in the ventral striatum ROI. (A) Illustration of the ventral striatum ROI encompassing bilateral putamen and nucleus accumbens. (B) Encoding of incentive in the ventral striatum was significantly stronger in the baseline condition, in which participants choked, than in the reappraisal condition. (C) The difference in the effect of incentive magnitude between the two conditions was associated with differences between the conditions in the degree of choking. Effects in panel (B) and (C) are in arbitrary units (a.u.).

Given that we previously demonstrated that the encoding of the monetary incentive in ventral striatal BOLD signal is associated with behaviour in the motor task (Chib *et al*., 2012, 2014), this region was an a priori region of interest (ROI) in the current study (see Methods for details of ROI definition). To implement the ROI analysis we regressed the average BOLD time course from this ROI (see Figure 4A) on the same design matrix used in the voxel-wise analysis of the BOLD signal across the whole brain. This analysis also demonstrated that BOLD in the ventral striatum at the time of initial incentive presentation [t(35) = 5.57, *P* < 1e-6, one-sided] increased with increasing incentive magnitude. Furthermore, this effect was greater in the baseline condition, in which participants choked, than in the reappraisal condition [t(35) = 2.28, *P* = 0.03], two-sided, see Figure 4B.

### ROI analysis: relationship between ventral striatum signal and behavioral sensitivity to choking

In order to further interrogate the difference between the conditions in incentive coding during the time of incentive presentation, we regressed this difference on the behavioral differences between the conditions in their degree of choking. We found that this difference in neural sensitivity to incentive magnitude in the ventral striatum was indeed associated with the difference in choking between the conditions, such that greater sensitivity to incentive in a condition was associated with greater choking in that condition [t(35) = −2.46, *P* = 0.02, see Figure 4C]. These results demonstrate that individual differences in the degree of attenuation of the ventral striatum response to incentive were correlated with the effectiveness of the reappraisal strategy in reducing choking.

We found no correlation between the differences in neural sensitivity to incentive magnitude in the ventral striatum to self-rated reappraisal scores that participants provided.

### Whole brain analysis: main effect of reappraisal at the times of incentive presentation and motor task performance

In order to test for a main effect of engagement of the reappraisal strategy on BOLD responsiveness to the task, we tested for differences between the baseline and reappraisal conditions in average BOLD activity (i.e. for an overall difference in activity pooled across incentive levels). We found no significant differences between these conditions at our whole brain threshold at the time of incentive presentation or at the time of execution of the motor task.

### Effects of incentive and appraisal on sympathetic arousal

**Fig. 5:**
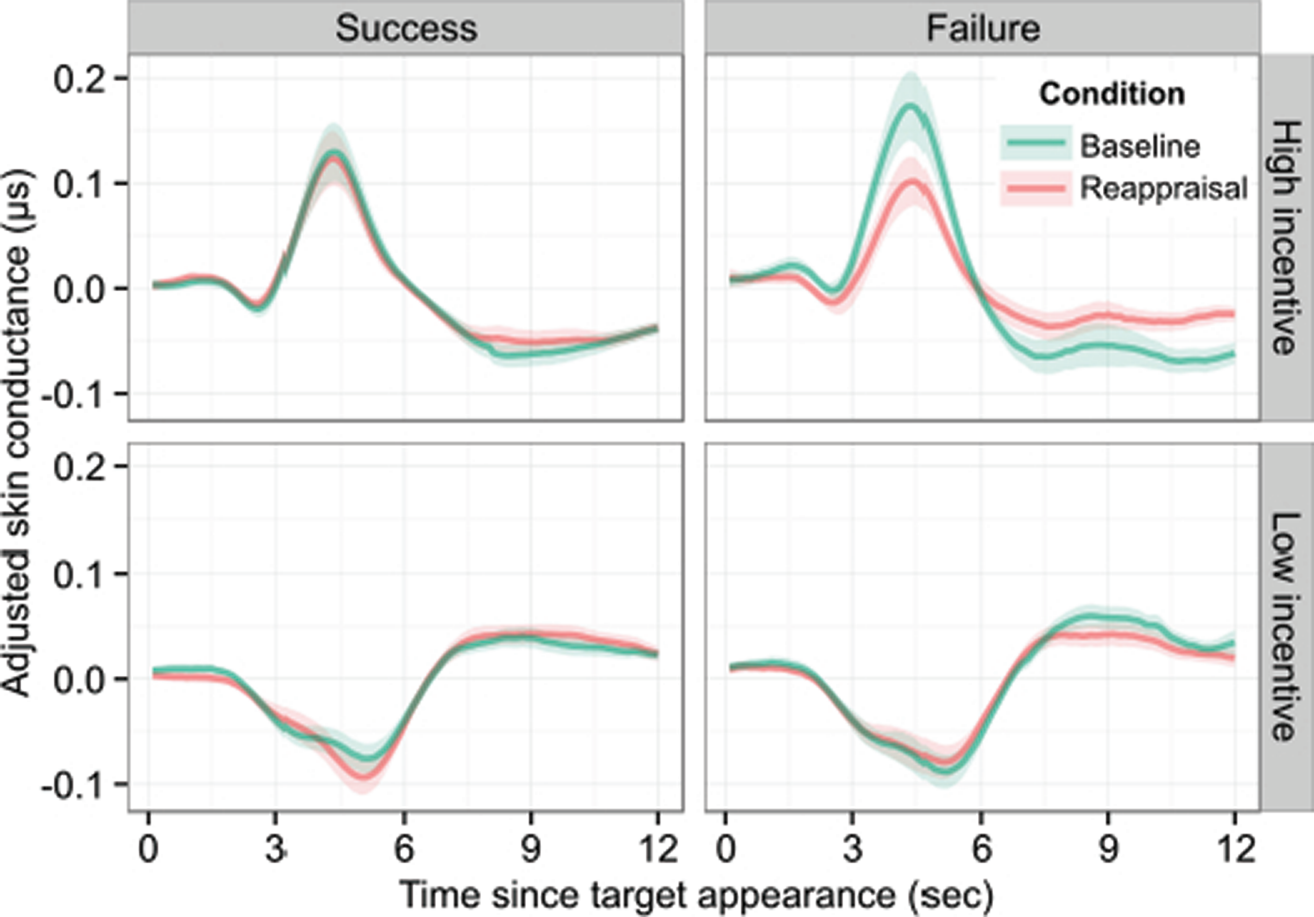
Effects of incentive magnitude on sympathetic arousal. Adjusted skin conductance aligned to the onset of the motor task. Skin conductance reflected the magnitude of the incentive with conductance increasing with increasing incentive. In addition, task failure in the baseline condition was accompanied by stronger encoding of high incentive relative to the reappraisal condition during the time of the motor task. In order to illustrate the effect of incentive at the time of motor task, each participant’s filtered and downsampled skin conductance data was adjusted for the estimated effects of all regressors in the design matrix, excluding those representing the main and interaction effects of incentive at the time of the motor task. The high incentive data is taken from $100 trials, while the low incentive data is taken from $0. Line represents mean and shadow represents SEM.

We also analysed participants’ skin conductance as they performed the task, using a general linear convolutional model, akin to those used in the analysis of fMRI data (Bach and Friston, 2013). This showed that skin conductance was responsive to task events such as the initial presentation of the incentive [t(37) = 6.02, *P* = 5.90e-7], and the period during which participants executed the motor task [t(37) = 3.17, *P* = 3e-3; see Supplementary Table S3]. The magnitude of each of these responses was modulated by the magnitude of the incentive available on that trial. Furthermore, we observed the hypothesised effect of the interaction of the magnitude of the incentive, participant’s performance (success or failure) and the condition during the performance of the motor task [t(37) = −2.54, *P* = 0.02], such that on failed trials, the effect of the incentive on skin conductance was significantly stronger in the baseline condition, in which participants choked, than in the reappraisal condition (see Figure 5). This effect is consistent with a claim that choking is associated with a state of sympathetic hyperarousal.

We conducted a follow-up analysis to determine whether this interaction effect was a result of a significant increase in skin conductance in response to high incentives on failed trials, a significant decrease for low incentives, or both (see Supplementary Table S4). We did this by creating separate indicator variables for trials from each combination of condition (baseline/reappraisal), performance (successful/unsuccessful) and incentive level (High = $100, Low = $0/$25/$50/$75). Comparisons of the resulting parameter estimates demonstrated that skin conductance on failed, high incentive trials in the baseline condition was significantly greater than on successful, high incentive baseline trials [t(37) = 2.60, *P* = 0.01] and significantly greater than failed high incentive reappraisal trials [t(37) = 3.32, *P* = 0.002]. The response to low incentive failed trials in the baseline and reappraisal conditions did not significantly differ [t(37) = −0.73, *P* = 0.47], which is consistent with a specific association between hyperarousal and choking rather than a more general association between hyperarousal and poor task performance. We obtain the same qualitative results when high incentive trials were defined as those on which the participant played for $75 or $100 (see Supplementary Table S4). Thus, we conclude that sympathetic arousal was indeed selectively increased when participants’ performance failed at high incentives when participants interpreted the incentive as a potential monetary gain, the condition that was specifically associated with choking.

A follow-up analysis did not find a significant relationship between this effect of the interaction of condition, incentive and performance on skin conductance at the time of the motor task with the differences between the conditions in neural sensitivity to incentive magnitude which we observe in the ventral striatum at the time of incentive presentation.

## Discussion

In this study we investigated the behavioral, neural and physiological effects of a novel intervention for choking under pressure, which targets the representation of the incentive with a cognitive reappraisal strategy. We confirmed that when participants interpreted the incentive as a potential monetary gain they choked under pressure; that is, their performance at the highest level of incentive was significantly worse than their performance at its peak. However, when they reappraised the incentive as a potential monetary loss, this choking effect was significantly reduced.

Our main behavioral prediction was that reinterpretation of the positive monetary incentive for successful performance as a potential monetary loss would selectively rescue highly loss-averse participants from choking under pressure, as suggested by previous findings (Chib *et al*., 2012, 2014). While we did observe a main effect of reappraisal on choking, which is arguably a more general and important result, unexpectedly, we found that loss aversion did not significantly interact with the appraisal condition to influence task performance. One potential explanation for this is that in both conditions of the present study, and unlike in Chib *et al*. (2012, 2014), participants were explicitly instructed to reflect on the consequences of gaining and losing the incentive presented on each trial. By encouraging this approach, as opposed to allowing for differences in cognitive strategies to manifest as a result of individual differences in incentive sensitivities and loss aversion, we may have reduced any intrinsic interindividual effects of loss aversion. Thus, our task manipulation may have had the unexpected, but potentially useful, consequence of minimizing any effect of loss aversion, such as those found in previous studies using this same behavioral task.

By temporally separating the presentation of the monetary incentive from the performance of the motor task, the task design allowed us to isolate distinct components of neural and physiological processing that were modulated by the reappraisal strategy. We hypothesized that if the behavioral effects of the reappraisal strategy derive from internal reframing of the incentive, we would expect to find modulation of the BOLD representation of incentive magnitude during reappraisal. Our findings are consistent with this account, with reappraisal of the monetary incentive as a potential monetary loss causing diminished neural encoding of the magnitude of the incentive in ventral striatum. Furthermore, we show that individual differences in the magnitude of this weakening in the ventral striatum predicted individual differences in choking between the reappraisal and baseline conditions, such that those with greater decreases in BOLD sensitivity to incentive had greater reductions in choking. These results reaffirm the role of the ventral striatum in responding to incentives (Knutson *et al*., 2001a; Knutson *et al*. 2001b; Seymour *et al*., 2007; Chib *et al*., 2012, 2014) and the influence that cognitive strategies can have on such responses (Delgado *et al*., 2008), but extend our understanding of the contribution of this region to behavioral choking by demonstrating that activity changes induced in this region due to different cognitive strategies are associated with behavioral changes in choking susceptibility. While strong claims about causality are beyond the remit of a correlative technique such as fMRI, these findings are consistent with a fundamental relationship between incentive-related activity in the ventral striatum and susceptibility to choking.

In contrast to previous neuroimaging studies (Ochsner *et al*., 2002, 2004; van Reekum *et al*., 2007; Ochsner and Gross, 2008; Wager *et al*., 2008), reappraisal was not associated with greater activation of prefrontal cortex. The recruitment of prefrontal cortical regions during reappraisal is suggested to reflect the exertion of cognitive control over subcortical emotional centers (Ochsner and Gross, 2008), a proposal that has been supporting by a finding that subcortical structures activated during reappraisal mediate the influence of lateral prefrontal regions on reappraisal (Wager *et al*., 2008). One possible explanation for the divergence of the present findings from the prior literature is due to potential differences in the regulatory strategy applied by participants in this study. Here, participants were instructed in the form of reappraisal they should apply, but were unaware of the intended behavioral consequences of the reappraisal strategy. This differs from previous studies of reappraisal, where the instructions and nature of the stimulus communicate to the participant the intended effects of the intervention on emotional behaviors and self-reported emotional experience. The neural and behavioral effects obtained from such manipulations may therefore reflect a combination of both the consequences of cognitive reappraisal of the stimulus, and the direct intentional inhibition of the behavioral and emotional indices of the stimulus. This would be consistent with neuroimaging literature on response inhibition, which reports the recruitment similar regions of prefrontal cortex to those reported by previous studies involving reappraisal (Konishi *et al*., 1999; Aron *et al*., 2004; Goldin *et al*., 2008).

The model-based analysis of skin conductance revealed that reappraisal was associated with a moderation of sympathetic arousal. Firstly, skin conductance rose significantly during the execution of the motor task, with an amplitude that was proportional to the size of the incentive. In the baseline condition, in which participants exhibited choking at high levels of incentive, we found that the effect of incentive on skin conductance was heightened when participants’ performance failed. *Post hoc* analyses confirmed that this effect was driven by greater skin conductance when playing for high monetary incentive, rather than reduced skin conductance when playing for low incentive. Taken together, the sympathetic response to incentive was lowest when participants played for low incentive, was greater when participants played for high incentive and succeeded and greatest when participants played for high incentive and failed; a pattern that is reminiscent of the relationship between performance and arousal described by the Yerkes–Dodson law (Yerkes and Dodson, 1908). These results build upon recent related work by Watanabe *et al*. (2018), who explore the relationship between incentive, performance, BOLD activity and an alternative measure of physiological arousal, pupil dilation. In particular, they (Watanabe *et al*., 2018) show that in the period immediately before task execution, pupil dilation was driven, in part, by incentive magnitude. The results of our analysis of skin conductance are consistent with this finding and we extend it significantly by demonstrating that physiological arousal is elevated specifically when performance fails at high incentive, and that this hyperarousal occurs during task performance. Although an association between hyperarousal and choking has been proposed (Ariely *et al*., 2009; Balk *et al*., 2013), this finding is to our knowledge, the first empirical evidence of heightened sympathetic arousal during choking.

Furthermore, we find that this sympathetic hyperarousal effect was abolished by reappraisal of the incentive; that is, failures of performance under high incentive during reappraisal were indistinguishable from successes under high incentive. This suggests that the heightened sympathetic response during failed performance for large incentives in the baseline condition is specifically associated with choking, given that we find neither choking nor an effect on skin conductance during reappraisal.

Choking under pressure in particular domains has been attributed to the interference of specific cognitive responses evoked by high incentives. One such account is that high incentives occupy working memory capacity that would otherwise be available for task execution (Wine, 1971). Accordingly, choking in tasks that require significant working memory resources, such as mathematical reasoning, have been shown to be accounted for by such a distraction mechanism (Beilock *et al*., 2004; Beilock and Carr, 2005; Gimmig *et al*., 2006; Markman *et al*., 2006; Beilock and DeCaro, 2007). An alternative account (Baumeister, 1984; Lewis and Linder, 1997) suggests that high incentives increase the degree of attention that is paid to the task, which paradoxically disrupts smooth proceduralised execution of the task and gives rise to poor performance. Highly practiced sensorimotor tasks such as golfing (Lewis and Linder, 1997; Beilock and Carr, 2001) and baseball (Gray, 2004), which rely on highly stereotyped, automatic responses that are generated without conscious attention appear to be particularly affected by this incentive-induced response.

Previous interventions have therefore targeted the specific responses believed to give rise to choking in a particular task, by reducing the working memory load induced by high incentives in reasoning tasks (Ramirez and Beilock, 2011; Balk *et al*., 2013) or by habituating participants to explicit scrutiny of their own performance in motor tasks (Beilock and Carr, 2001).

In contrast, by targeting the perception of the incentive itself, rather than a specific disruptive response evoked by the incentive, the mechanism of the present intervention differs categorically from those of previous interventions. The neural data indicates that the present approach was successful in this regard, with the neural effect of the intervention being to alter the representation of the incentive when the incentive was presented to the participant, before they performed the motor task. This manipulation of the processing of the incentive also influenced the downstream effects of the incentive, with a subsequent reduction in both the autonomic hyperarousal response to high incentives and choking under pressure. Furthermore, because this intervention targets the incentive directly, it may have the advantage of being applicable to a greater range of domains than previous interventions, which are limited to domains in which choking is caused by the effect of the incentive that they target. However, further work is required to determine whether the effects we identify are unique to reappraising the monetary gain as a loss, or can be obtained by alternative forms of reappraisal.

In summary, we validate a novel intervention that successfully abolishes performance decrements under high incentives in a skilled motor task, and identify its underlying neural and physiological substrates. Although further testing is required to determine the generality of this intervention to other types of task, by targeting the representation of the incentive, reappraisal may prove to be a highly flexible intervention for choking under pressure.

## Funding

This work was funded by grant NSF 1062703 from the National Science Foundation to J.P.O.D.

## Supplementary Material

Supplementary Data
