# Supplementary figures and images for: Reappraisal of incentives ameliorates choking under pressure and is correlated with changes in the neural representations of incentives
Source: Soc Cogn Affect Neurosci. 2018 Nov 27;14(1):13–22. doi: 10.1093/scan/nsy108 (PMC6318472; doi:10.1093/scan/nsy108)

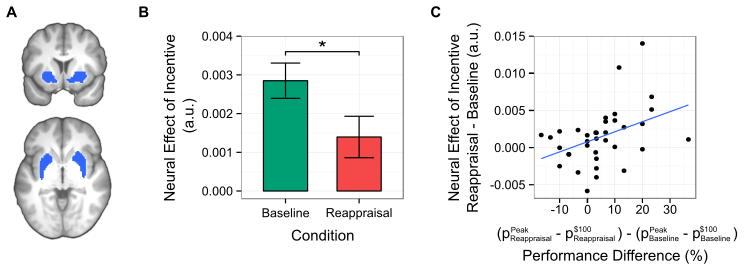

Supplement: Supplementary Data [file nsy108_supp.zip › scan-18-218-File005.png]

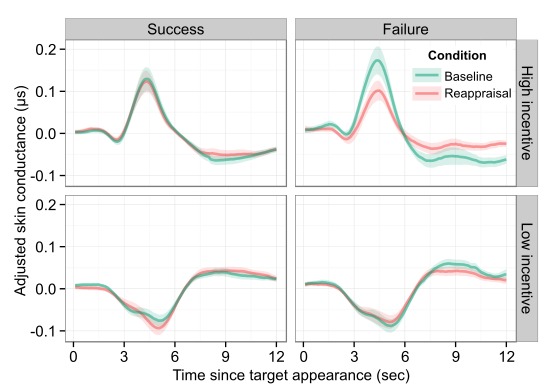

Supplement: Supplementary Data [file nsy108_supp.zip › scan-18-218-File006.png]

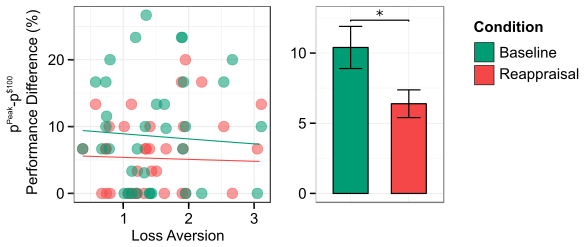

Supplement: Supplementary Data [file nsy108_supp.zip › scan-18-218-File003.png]

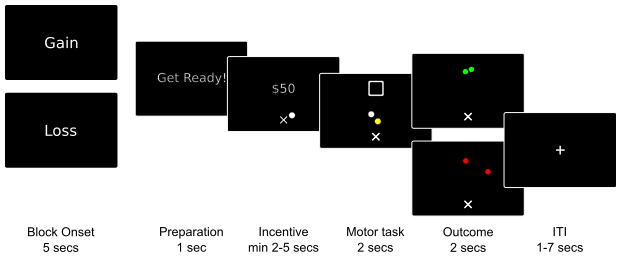

Supplement: Supplementary Data [file nsy108_supp.zip › scan-18-218-File002.png]

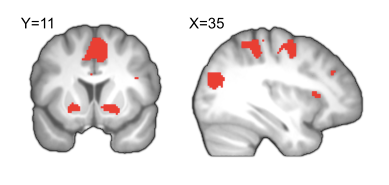

Supplement: Supplementary Data [file nsy108_supp.zip › scan-18-218-File004.png]
